# The Role of Endosomal Escape and Mitogen-Activated Protein Kinases in Adenoviral Activation of the Innate Immune Response

**DOI:** 10.1371/journal.pone.0026755

**Published:** 2011-10-27

**Authors:** Jeffrey S. Smith, Zhili Xu, Jie Tian, Donna J. Palmer, Philip Ng, Andrew P. Byrnes

**Affiliations:** 1 Division of Cellular and Gene Therapies, Food and Drug Administration Center for Biologics Evaluation and Research, Bethesda, Maryland, United States of America; 2 Molecular and Human Genetics, Baylor College of Medicine, Houston, Texas, United States of America; French National Centre for Scientific Research, France

## Abstract

Adenoviral vectors (AdV) activate multiple signaling pathways associated with innate immune responses, including mitogen-activated protein kinases (MAPKs). In this study, we investigated how systemically-injected AdVs activate two MAPK pathways (p38 and ERK) and the contribution of these kinases to AdV-induced cytokine and chemokine responses in mice. Mice were injected intravenously either with a helper-dependent Ad2 vector that does not express viral genes or transgenes, or with the Ad2 mutant *ts*1, which is defective in endosomal escape. We found that AdV induced rapid phosphorylation of p38 and ERK as well as a significant cytokine response, but *ts*1 failed to activate p38 or ERK and induced only a limited cytokine response. These results demonstrate that endosomal escape of virions is a critical step in the induction of these innate pathways and responses. We then examined the roles of p38 and ERK pathways in the innate cytokine response by administering specific kinase inhibitors to mice prior to AdV. The cytokine and chemokine response to AdV was only modestly suppressed by a p38 inhibitor, while an ERK inhibitor has mixed effects, lowering some cytokines and elevating others. Thus, even though p38 and ERK are rapidly activated after i.v. injection of AdV, cytokine and chemokine responses are mostly independent of these kinases.

## Introduction

Intravenous administration of high doses of non-replicating AdV causes a rapid innate immune response characterized by elevated cytokines and chemokines [1]–[4]. Inhibiting these innate immune responses could substantially improve the safety of AdV, and therefore it is important to identify drugs that block AdV-induced signaling pathways. However, not enough is known about the signal transduction cascades that are induced by AdV *in vivo*, nor whether targeting these individual signaling pathways might broadly suppress the innate immune response to AdV.

Importantly, it is known that blocking endosomal escape of virions prevents much of the innate response to AdV [5]–[7]. This implies that the sensors and signaling pathways of the innate immune system are triggered during or after endosomal escape of virions, and that these pathways may primarily be located intracellularly. MAPKs are intracellular signaling proteins that use phosphorylation cascades to generate cellular responses to environmental stimuli [8]. There are several classes of mammalian MAPKs, including extracellular signal-regulated kinase 1 and 2 (ERK), c-jun N-terminal kinase (JNK) and p38 MAPK, all of which are activated following phosphorylation by MAPK kinases (MAPKK). The MAPKK are in turn phosphorylated by MAPKK kinases. The ERK signaling cascade consists of Raf:MEK:ERK and can be activated by many stimuli, including viral infection and cytokines [8]. The p38 pathway can be activated by a variety of receptors, including cytokine receptors and toll-like receptors (TLRs), a family of well-studied pathogen recognition receptors [9], [10]. One important consequence of MAPK pathway activation is the stimulation of transcription factors involved in cytokine synthesis [11], [12], and the p38 pathway in particular is being targeted in clinical trials to treat inflammatory diseases [8], [12]–[14].

A number of studies have shown that AdVs activate p38 and ERK *in vitro*, and that these kinases are important for the induction of certain cytokines and chemokines by cultured cells [5], [15]–[17]. *In vivo*, intravenous administration of AdV activates ERK within 15 minutes in the mouse liver, in part by signaling through Toll-like receptor 2 via the downstream adapter molecules MyD88 and TRIF [18], [19]. However, it remains unclear whether p38 or ERK play any essential role in mediating the innate cytokine response *in vivo*, nor whether specific kinase inhibitors could be useful for broadly suppressing innate immune responses to AdV.

In this study, we evaluated activation of MAPK pathways and cytokine/chemokine responses after injecting mice i.v. with a replication-deficient helper-dependent Ad2 virus (HDAd2), and compared the response to a mutant Ad2 virus, *ts*1. When *ts*1 is grown at the non-permissive temperature of 39°C, the resulting virions are unable to escape from endosomes during entry [20], [21]. Thus, we were able to examine whether innate responses were triggered before or after endosomal escape of virions. We subsequently investigated how *in vivo* inhibition of p38 and ERK pathways with kinase inhibitors affected the innate inflammatory response to AdV.

## Results

### The *ts*1 mutant virus induces an attenuated cytokine/chemokine response

**Figure 1:**
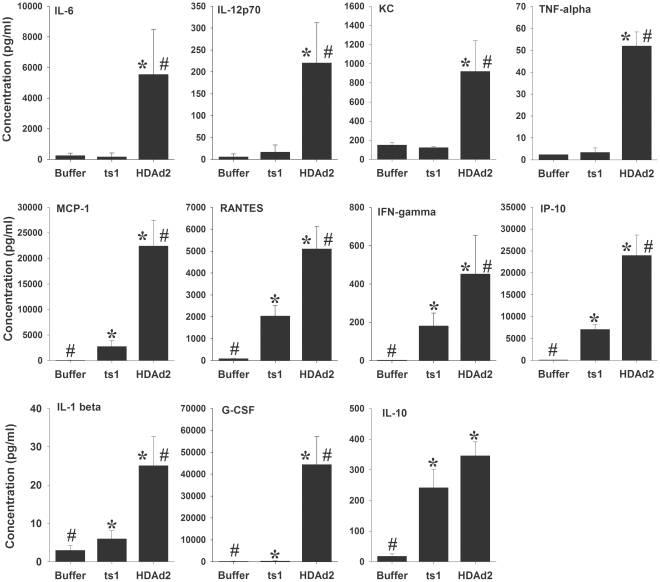
The *ts*1 virus induces an attenuated cytokine/chemokine response. Mice were injected i.v. with buffer, *ts*1 or HDAd2 at 5×10^12^ vp/kg, and serum was collected six hours after injection. Compared to buffer control animals, HDAd2 induced a significant increase in the levels of 11 cytokines and chemokines. In contrast, *ts*1 had a significantly attenuated response with all chemokines/cytokines except IL-10, which was not detectably different from the IL-10 response induced by HDAd2. 5 mice/group. * = *p*<0.05 vs. buffer control mice; # = *p*<0.05 vs. mice that received *ts*1 (ANOVA, Holm-Sidak).

To determine if endosomal escape is required for Ad to trigger the innate inflammatory response, we injected mice i.v. with buffer or 5×10^12^ viral particles (vp) per kg of either HDAd2 or *ts*1 grown at the non-permissive temperature. The HDAd2 vector that we used expresses no viral gene or transgenes, which allowed us to examine innate immune responses triggered by the virion components alone. Because neither HDAd2 nor *ts*1 expresses genes, we were able to avoid any possible pro-inflammatory effects that might be caused by expression of transgenes or viral genes. Administration of HDAd2 induced a significant elevation of 11 cytokines and chemokines in the serum at 6 h, when compared to mice receiving buffer only (Figure 1). In contrast, we found that *ts*1 induced a significantly attenuated innate cytokine response, failing to induce significant levels of IL-6, IL-12 p70, KC or TNFα. When compared to HDAd2, *ts*1 induced partial elevation of MCP-1, RANTES, IFNγ, IP-10, IL-1β and G-CSF, and fully induced IL-10. The serum level of GM-CSF was not detectably altered by HDAd2 or *ts*1 (not shown).

### ERK and p38 MAPKs are activated *in vivo* by HDAd2 but not *ts1*

**Figure 2:**
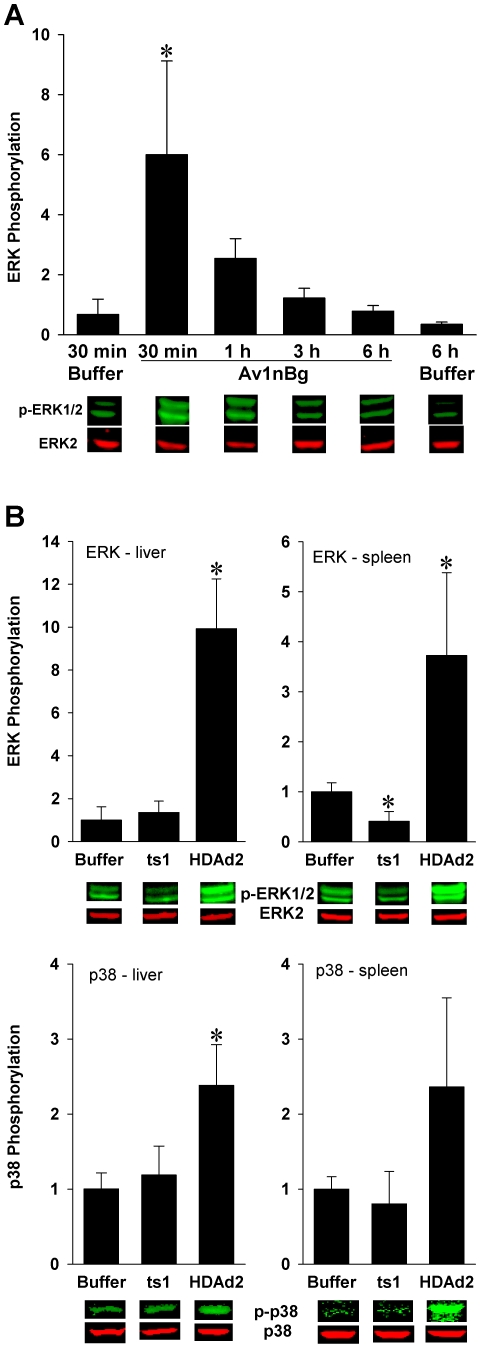
Activation of ERK and p38 by AdV in mouse liver and spleen. Phospho-ERK (p-ERK1/2) and phospo-p38 (p-p38) were quantitated by Western blot using phospho-specific antibodies. A. Av1nBg, injected i.v. at 5×10^12^ vp/kg, induced a rapid phosphorylation of ERK in the mouse liver, with a peak appearing at 30 min. * = *p*<0.05 vs. 30 min buffer control mice (ANOVA, Holm-Sidak). B. HDAd2 significantly induced phosphorylation of ERK in the liver and spleen and p38 in the liver at 30 minutes, but *ts*1 did not. Data are normalized to the buffer control group. 5 mice/group. * = *p*<0.05 vs. buffer control mice (ANOVA, Holm-Sidak).

We performed a series of experiments to examine the activation of ERK and p38 in the liver and spleen after administration of AdV. Appledorn *et al.* [18] have previously demonstrated a rapid phosphorylation of ERK in murine liver following i.v. administration of replication defective Ad5 vectors. We confirmed that i.v. injection of the Ad5 vector Av1nBg induces phosphorylation of ERK in the liver and we determined the kinetics of this response. Mice were injected with buffer or Av1nBg at 5×10^12^ vp/kg and liver lysates were evaluated by Western blot for phosphorylation of ERK. We observed a significant elevation in ERK phosphorylation at 30 min after Av1nBg (Figure 2A). ERK phosphorylation declined with time and was not significantly different than buffer control mice at times 3 hours or more after vector administration. Therefore, we chose a 30 minute time point for further experiments.

We found that HDAd2 elevated phosphorylation of both ERK and p38 in the liver and spleen (Figure 2B), although phosphorylation of p38 in the spleen did not reach significance in all experiments. In contrast, *ts*1 did not cause any elevation of ERK or p38 phosphorylation. The negative results with *ts*1 indicate that ERK and p38 pathways are triggered during or after endosomal escape of AdV.

### Inhibition of ERK or p38 pathways with kinase inhibitors

Because of the correlation that we observed between MAPK activation and cytokine/chemokine responses in the previous experiments, we used kinase inhibitors to test whether activation of the p38 or ERK pathways played any essential causal role in the cytokine and chemokine response to HDAd2. We selected two kinase inhibitors, PD0325901 and SB239063, based upon their demonstrated ability to selectively inhibit ERK and p38 pathways, respectively [22], [23]. Functionally, PD0325901 inhibits the enzymatic activity of MEK, which is the MAPKK responsible for phosphorylation of ERK. In contrast, SB239063 directly inhibits p38 kinase activity. The doses we selected for oral administration were based on the previously demonstrated pharmacological activity of these inhibitors in rodents [22], [23].

**Figure 3:**
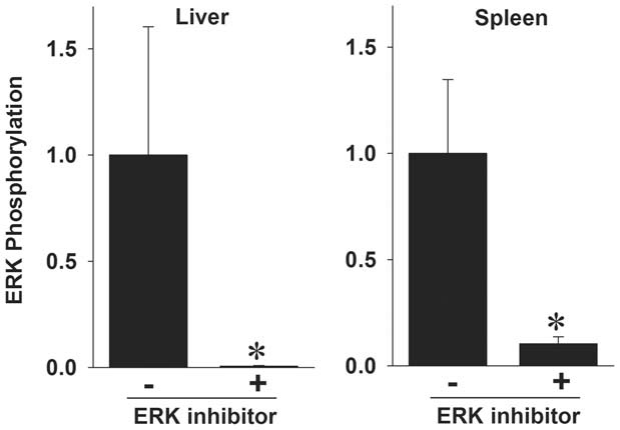
The MEK inhibitor PD0325901 significantly blocks AdV-induced phosphorylation of ERK in the liver and spleen. Mice were orally administered 10 ml/kg of vehicle (0.5% methylcellulose) or 100 mg/kg of a MEK inhibitor (PD0325901), and all mice were injected i.v. with HDAd2 60 minutes later. Livers and spleens were collected 30 minutes after HDAd2 for Western blot. Data are normalized to the vehicle control group. 4 mice/group * *p*<0.01 for vehicle control vs. PD0325901 (*t*-test).

We first evaluated the ability of PD0325901 to block the *in vivo* phosphorylation of ERK induced by AdV. Mice were dosed orally with either buffer (0.5% methylcellulose) or PD0325901 one hour before administering HDAd2. Livers and spleens were collected 30 minutes after AdV and lysates evaluated for ERK phosphorylation. When mice received PD0325901 prior to HDAd2, relative phosphorylation of ERK in the liver and spleen was significantly reduced (Figure 3), demonstrating the effectiveness of PD0325901 *in vivo*. We did not examine the *in vivo* inhibitory activity of SB239063 in a similar manner because this compound inhibits p38 activity, not p38 phosphorylation. However, we were able to demonstrate a significant *in vivo* inhibitory effect of SB239063 on LPS-induced cytokine responses (below).

**Figure 4:**
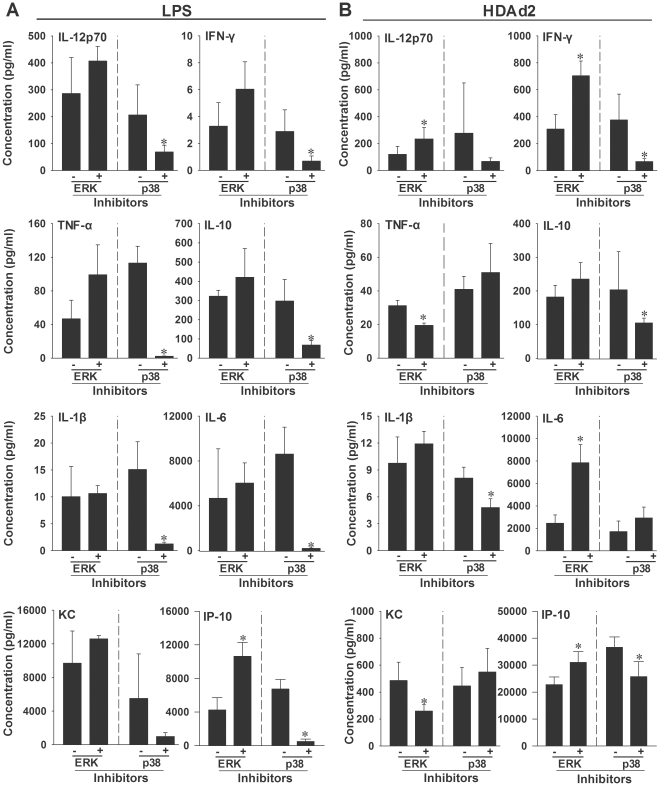
Effect of kinase inhibitors on the serum cytokine response in mice receiving HDAd2 or LPS. Mice were orally administered either 8 ml/kg buffer, 100 mg/kg of the MEK (ERK pathway) inhibitor PD0325901 or 40 mg/kg of the p38 inhibitor SB239063. Sixty minutes later mice were injected i.v. with 5×10^12^ vp/kg HDAd2 or i.p. with 500 µg/kg LPS. Serum was collected at either 2 hours (LPS) or six hours (HDAd2) and evaluated for cytokine levels. A. The cytokine response to LPS was unaltered by inhibition of the ERK pathway with the exception of IP-10, which was signficantly elevated (left panel). In contrast, inhibition of the p38 pathway significantly reduced the response to LPS for all the tested chemokines/cytokines except KC (right panel). B. Inhibition of the ERK pathway altered the cytokine/chemokine response to HDAd2 (left panel), significantly inhibiting the serum levels of TNFα and KC and significantly elevating the expression of IL-12p70, IFNγ, IP-10 and IL-6. Pretreatment with the p38 inhibitor (right panel) significantly decreased the serum levels of IFNγ, IL-10 and IL-1β. The buffer controls were 0.5% methylcellulose and 0.5% acidified tragacanth for the ERK and p38 inhibitors, respectively. 3–5 mice/group, * = *p*<0.05 vs. corresponding buffer control (*t*-test).

To determine if these two kinase inhibitors could affect the serum cytokine response induced by AdV, mice were orally administered buffer, PD0325901 or SB239062, followed by HDAd2 or LPS. A 500 µg/kg dose of LPS was used as a positive control because this is known to activate the ERK and p38 pathways and elevate cytokines in rodents [22], [24]. The MEK inhibitor PD0325901 did not significantly inhibit the cytokine/chemokine response to LPS (Figure 4A). In contrast, the p38 inhibitor SB239063 markedly suppressed the response to LPS (Figure 4A). Thus, the LPS-induced cytokine/chemokine response is considerably more dependent on the p38 pathway than on the ERK pathway.

When mice were pre-treated with the MEK inhibitor PD0325901 prior to HDAd2, the levels of KC and TNFα were significantly reduced (Figure 4B). Interestingly, HDAd2 induction of IL-6, IL-12p70, IP-10 and IFNγ was significantly increased by PD0325901, suggesting that the ERK pathway negatively regulates production of these cytokines. Pre-treatment with the p38 inhibitor SB239063 modestly suppressed the cytokine response to HDAd2, significantly decreasing levels of IL-10, IFNγ, IP-10 and IL-1β (Figure 4B). Other cytokines and chemokine responses were not significantly affected, however, including TNFα, IL-6, IL12p70 and KC (Figure 4B). Thus, targeting p38 had only modest and selective effects on the cytokine and chemokine response to HDAd2, and targeting ERK actually made some responses worse.

## Discussion

The innate immune response to AdV involves the activation of multiple sensing and signaling pathways, culminating in the expression of inflammatory cytokines and chemokines. Depending on the cell type, AdV can be sensed by as many as three different classes of pathogen recognition receptors: TLRs, NOD-like receptors and RIG-I-like receptors [3], [4], [18], [25], [26]. These receptors activate signal transduction pathways that ultimately induce cytokines and chemokines, but these signaling pathways have not been extensively studied *in vivo* after systemic injection of AdV. In this study we have shown that i.v. injection of a helper-dependent AdV, devoid of any viral genes or transgenes, activates the p38 and ERK pathways in mice. Using the mutant virus *ts*1 we also found that that endosomal escape is required for activation of these MAPK pathways and full expression of inflammatory cytokines and chemokines. In spite of this correlation, when we blocked activation of MAPKs we found that p38 and ERK were not essential for most of the innate cytokine and chemokine response to AdV.

*In vitro* studies have demonstrated that endosomal escape of virions is a critical trigger for cytokine and chemokine upregulation [5], [6]. There is evidence that various innate immune sensors detect both lysis of vesicles by AdV as well as subsequently-exposed viral material such as AdV DNA. For example, the NLRP3 inflammasome is a key sensor of vesicle lysis, and AdV is able to disrupt lysosomes and activate NLRP3, but *ts*1 is not [27]. *In vivo*, Di Paolo *et al.* [7] found that while *ts*1 was taken up normally by marginal zone macrophages in the mouse spleen, it was attenuated in inducing certain early cytokine and chemokine responses. Fejer *et al.* [6] also found that *ts*1 fails to induce type I interferon in mice. In addition to *ts*1's poor ability to induce cytokines and chemokines, our *in vivo* studies have shown that *ts1* does not trigger a variety of other inflammatory and pathologic responses such as complement activation and Kupffer cell killing [28], [29].

In the current study, we screened a wide panel of cytokines and chemokines and demonstrated that, compared to HDAd2, *ts*1 is significantly attenuated in stimulating the secretion of many innate inflammatory cytokines and chemokines into the blood stream 6 hours post i.v. injection. However, we found that a subset of cytokines were partially elevated by *ts*1, indicating activation of some innate immune responses even without viral penetration. A previous study showed that *ts*1 can promote mRNA expression of IL-1α in the mouse spleen at 30 minutes and small elevations of some cytokines at 60 minutes [7]. Interestingly, the IL-1α response in the spleen was found to be dependent on interactions between AdV and integrins [7], and integrins have also been reported to be involved in AdV-induced keratitis [30]. These results suggest the possibility that activation of integrins might contribute to the ability of *ts*1 to induce cytokine and chemokine responses.

MAPK pathways activate transcription factors that control cytokine production [8]. Both ERK and JNK pathways are thought to be important in activation of the transcription factor AP-1, which regulates many cytokine genes, and the p38 pathway is involved in the induction of multiple pro-inflammatory cytokines [12]. Although we were unable to detect induction of JNK by AdV in the liver or spleen (data not shown), JNK has been shown to play a critical role in the type I interferon response to AdV both *in vitro* and *in vivo* [6]. Regarding the role of ERK, this MAPK is activated by AdV both *in vitro* and *in vivo* [5], [15], [17], [18]. Regarding p38, AdV activates p38 in cultured cells [5], [16], but whether p38 is activated by AdV has not previously been measured *in vivo*.

*In vitro* studies have indicated a correlation between endosomal escape of AdV and MAPK activation. Tibbles *et al.* [5] demonstrated that blocking endosomal acidification inhibited p38 and ERK phosphorylation in epithelial cells, and Suomalainen *et al.* [16] found that *ts*1 was deficient in stimulating p38 in HeLa cells. Because the kinase response to *ts*1 had not previously been examined *in vivo*, we quantitated ERK and p38 phosphorylation in mouse liver and spleen. AdV induced phosphorylation of both ERK and p38 within 30 min *in vivo*. In contrast, we found that *ts*1 failed to elevate the phosphorylation of either of these two MAPKs, indicating a correlation among endosomal escape, activation of MAPK pathways and the cytokine and chemokine response to AdV.

To determine whether p38 or ERK plays any critical role in the AdV-induced cytokine response, we administered two specific MAPK inhibitors to mice. The ERK pathway inhibitor, PD0325901, is a specific non-ATP competitive inhibitor of MEK, with well described pharmacokinetics, that blocks the phosphorylation of ERK *in vivo* [23]. We demonstrated that PD0325901 was effective in blocking HDAd2-induced ERK phosphorylation. The p38 inhibitor SB239063 blocks the kinase activity of p38 and has been shown to inhibit the production of LPS-induced cytokines [22]. We confirmed the *in vivo* activity of SB239063 in our experiments by showing that it markedly suppressed the cytokine and chemokine response to LPS.

We found that inhibition of MEK did not suppress the cytokine response to LPS and only inhibited the induction of one AdV-induced cytokine (TNF-α) and one AdV-induced chemokine (KC). Interestingly, blocking ERK phosphorylation actually enhanced AdV induction of IL-6, IL-12p70, IP-10 and IFNγ. This finding suggests that the ERK pathway may contribute to feedback inhibition of these cytokines. Our results on serum IP-10 at 6 h after AdV contrast with those of Tibbles *et al.* [5], who reported that a different MEK inhibitor partially suppressed IP-10 mRNA levels in liver at an early timepoint after AdV (1 h). These authors also found that a p38 inhibitor was partially suppressive for IP-10 mRNA. However, they reported that the effect of the p38 inhibitor did not persist beyond 1 h, and by 6 h the IP-10 mRNA response to AdV was similar regardless of whether a p38 inhibitor was used [5]. It is possible that the earliest steps of AdV-induced innate immune responses in certain organs are partially dependent on p38 or ERK, but redundant pathways eventually allow full peak serum levels of cytokine and chemokine.

When we examined the effect of a p38 inhibitor on the AdV-induced response, we found a partial but statistically-significant reduction in 4 of the 8 tested cytokines and chemokines. Clearly, the majority of the AdV-induced response did not require p38 activity. In contrast, the response to LPS was nearly eliminated by the p38 inhibitor, with a significant reduction in 7 of the 8 tested cytokines and chemokines and a non-significant reduction in the serum level of KC.

The discordance between the effectiveness of the p38 inhibitor at inhibiting the response to LPS, but not the response to AdV, highlights the complexity of the innate response to AdV. This makes sense in light of the known redundancy of innate immune sensors that can detect AdV. For example, the cytokine response to LPS is mediated through TLR4 [31], but studies on the innate response to AdV have demonstrated involvement of multiple TLRs including TLR2, TLR3, TLR4 and TLR9, each of which partially contributes to sensing AdV [18], [19], [32], [33]. In addition, a number of other innate sensors also detect AdVs, including NOD-like receptors and RIG-I-like receptors [4]. Thus, AdV activates a wider variety of sensors than LPS and the resulting network of responses is far more complex, with the implication that using drugs to target single pathways may only show limited effectiveness in decreasing the broad innate immune response to AdV.

In conclusion, although small-molecule drugs that interfere with MAPK pathways are thought to have promise as anti-inflammatory drugs, we found that neither the ERK nor p38 pathways played an essential role in the broad cytokine and chemokine response to AdV. Nevertheless, these results add important knowledge to our understanding of the roles of MAPKs *in vivo*. Given the complexity of the innate immune reaction to systemically injected AdV, it seems likely that one will need to target multiple pathways simultaneously to achieve meaningful suppression of the innate response to AdV.

## Materials and Methods

### Ethics Statement

All animal protocols and procedures were approved by the FDA CBER Animal Care and Use Committee (protocol #2003-11) in animal facilities accredited by the Association for Assessment and Accreditation of Laboratory Animal Care International. All experiments were performed according to institutional guidelines.

### Viruses

The helper-dependent Ad2 virus HDAd2Δ28E4 (HDAd2) was generated by rescuing the HDAd plasmid pΔ28E4 (Toietta et al., 2002) with the Ad serotype 2 helper virus Ad2LC8cCARP (Parks et al., 1999) in 116 cells as described elsewhere (Palmer and Ng, 2003). pΔ28E4 has a 28 kb genome that is composed of Ad5 derived inverted terminal repeats and a packaging signal, followed by two non-coding mammalian stuffer sequences and has no transgene [34]. The Ad2 mutant *ts*1 was grown at 39°C and characterized as previously described [28]. The particle:pfu ratio of *ts*1 was greatly elevated (1×10^5^), as expected. A replication-defective E1/E3-deleted Ad5 vector expressing nuclear-localized β-galactosidase (Av1nBg) was used for certain experiments [35].

Viruses were purified by double (for *ts*1 and Av1nBg) or triple (for HDAd2) CsCl ultracentrifugation as described previously [36], [37]. Ad concentration was measured spectrophotometrically by the OD260 method, with no detectable aggregation, as previously described [36], [38]. Endotoxin levels in all substances injected into mice were <0.15 EU/ml by the LAL method (Charles River Endosafe, Charleston SC).

### Animals

Male C57BL/6NCr or C57BL/6J mice were obtained from the National Cancer Institute (Frederick, MD, USA) or Jackson Labs (Bar Harbor, ME) respectively. The mice were maintained in our specific pathogen-free facilities, and used at 8–10 weeks of age. For some experiments the ERK inhibitor PD0325901 (100 mg/kg, Selleck Chemicals, Houston, TX), suspended in 0.5% aqueous methylcellulose (Sigma), or the p38 inhibitor SB239063 (40 mg/kg, Tocris Biosciences, Ellisville, MO), suspended in an aqueous solution of 0.5% tragacanth (Sigma) containing 0.05N HCl (acidified tragacanth), were administered per os. For per os administrations, awake mice were restrained by hand and buffer or inhibitors administered directly into the stomach at 10 ml/kg through a 20G, 1.5″, curved feeding needle with a 2.25 mm ball tip. LPS from *E. coli* 026:B6 (Sigma, St. Louis, MO) was prepared in phosphate buffered saline and administered i.p. at 0.5 mg/kg. For tail vein injections of AdV, awake mice were restrained in a cylindrical device and a dose of 5×10^12^ vp/kg was administered at 8.0 ml/kg over approximately 5 s. Five to 10 min prior to sacrifice, animals were anesthetized by intraperitoneal injection with 150 mg/kg ketamine and 30 mg/kg xylazine. For serum collections, blood was obtained by cardiac puncture. For Western blots, livers and spleens were removed, placed in microfuge tubes, immediately frozen on dry ice and transferred to −80°C freezer until lysed for Western blots as described below.

### Western Blots

Frozen liver and spleen fragments, weighing between 150–250 mg and 50–120 mg respectively, were homogenized on a Bullet Blender (Next Advance) in 400–500 µl chilled lysis buffer (20 mM Tris-HCL, pH 7.4, 1 mM EDTA and 150 mM NaCl) containing 1% Triton X-100. A protease inhibitor cocktail (Sigma #P8340) and phosphatase inhibitor cocktail 1 (Sigma #P0044) were added to the lysis buffer at 1% just prior to use. Lysates were cleared at 12,000 g, 4°C, for 10 minutes, and protein concentrations determined (DC protein assay, Bio-Rad, Hercules CA) prior to storage at −80°C. For Western blotting, equal amounts of protein samples were run on polyacrylamide gels and transferred to nitrocellulose membranes. Membranes were probed with primary antibodies to ERK2 (Santa-Cruz sc-81458), p38 (Santa Cruz sc-7972), phosphorylated-ERK1/2 (Cell Signaling-Danvers, MA) #4370S or phosphorylated p38 (Cell Signaling) #4511 and detection performed using fluorescent secondary antibodies from Odyssey/LI-COR (Lincoln, Nebraska). Blots were scanned and bands quantified by fluorescent intensity using a LI-COR scanner. For data analysis, the amount of phosphorylated ERK1/2 was normalized to total ERK2 protein as described by Appledorn *et al.* [18]. Similarly, phosphorylated p38 was normalized to total p38.

### Cytokines and chemokines

Serum chemokine/cytokine concentrations were evaluated using singleplex and multiplexed assay kits from Meso Scale Discovery (MSD, Gaithersburg, MD) or ELISA kits from R&D systems (Minneapolis, MN). Specifically, serum was used in multiplex (7-plex mouse pro-inflammatory) kits from MSD to evaluate IFN-γ, IL-10, IL-12p70, IL-1β, IL-6, KC and TNF-α. Singleplex assays from MSD were used for MCP-1, GM-CSF and RANTES. When required due to exceeding upper limit of detection, an MSD singleplex was used for IL-6. MSD plates were evaluated on an MSD Sector Imager 2400, model 1250. Mouse Quantikine kits from R&D systems were used for detection of G-CSF and IP-10. In all cases samples were assayed in duplicate.

### Statistical analysis

In all figures, mean ± SD is shown. Data were log-transformed to equalize variances among groups, and then analyzed by either *t*-test or ANOVA, depending on the number of groups in the experiment. For ANOVA, post-hoc comparisons between control and test groups were made using the Holm-Sidak test (SigmaPlot 11.0, Systat Software, San Jose CA). Significance was defined as *p*≤0.05.
